# Effects of Transcatheter Mitral Valve Repair Using MitraClip^®^ Device on Sleep Disordered Breathing in Patients with Mitral Valve Regurgitation

**DOI:** 10.3390/jcm10153332

**Published:** 2021-07-28

**Authors:** Ayham Daher, Tobias Müller, Nikolaus Marx, Jörg Schröder, Mohammad Almalla, András P. Keszei, Sebastian Reith, Michael Dreher

**Affiliations:** 1 Department of Pneumology and Internal Intensive Care Medicine, University Hospital RWTH, 52074 Aachen, Germany; tobmueller@ukaachen.de (T.M.); mdreher@ukaachen.de (M.D.); 2 Department of Cardiology, Angiology and Internal Intensive Care Medicine, University Hospital RWTH, 52074 Aachen, Germany; nmarx@ukaachen.de (N.M.); jschroeder@ukaachen.de (J.S.); malmalla@ukaachen.de (M.A.); sebastian.reith@sfh-muenster.de (S.R.); 3 Center for Translational & Clinical Research Aachen (CTC-A), University Hospital RWTH, 52074 Aachen, Germany; akeszei@ukaachen.de; 4 Clinic for Cardiology and Angiology, St. Franziskus-Hospital, 48145 Münster, Germany

**Keywords:** mitral regurgitation, MitraClip, sleep disordered breathing, respiratory polygraphy, brain natriuretic peptide

## Abstract

Sleep disordered breathing (SDB) is common among patients with valvular heart disease, and successful valve surgery could reduce SDB severity. However, data about the effects of transcatheter mitral valve repair on SDB are scarce. Therefore, mitral regurgitation (MR) patients undergoing MitraClip-placement were prospectively enrolled. Before MitraClip-placement, daytime sleepiness and sleep quality were assessed using the Epworth Sleepiness Scale (ESS) and Pittsburgh Sleep Quality Index (PSQI), respectively; and all patients underwent SDB screening using five-channel respiratory polygraphy. After 3–6 months, patients had a similar reassessment including: ESS, PSQI, and respiratory polygraphy. 67 patients were included (77 ± 8years). Despite normal sleepiness scores, 41 patients (61%) had SDB with apnea-hypopnea-index (AHI) ≥ 15 h before MitraClip-placement, of whom only three patients had known SDB previously. Compared to patients without SDB, patients with SDB had similar sleepiness scores but higher NT-proBNP values at baseline (4325 vs. 1520 pg/mL, *p* < 0.001). At follow-up, there were significant AHI improvements among patients with SDB (*p* = 0.013). However, there were no significant sleepiness score changes. In conclusion, the prevalence of SDB among MitraClip candidates is very high even in those without daytime sleepiness. MR patients with SDB have higher NT-proBNP values, which may reflect a worse prognosis. MitraClip-placement may improve the underlying SDB, which could be an additional benefit of the procedure.

## 1. Introduction

Mitral regurgitation (MR) is a common valvular disorder, which can significantly impair or worsen cardiac function and reduces patients’ quality of life (QoL) mainly due to exertional breathlessness and decreased exercise tolerance [1,2]. Currently, symptomatic patients with MR are candidates for interventional edge-to-edge therapy of the anterior and posterior mitral leaflets using MitraClip device, which has been shown to significantly improve QoL and prognosis in carefully selected patients [3].

A growing body of evidence shows that sleep disorders are common among patients with cardiac disease, and the bidirectional relationship between both illnesses has been arousing a growing interest in the last years [4,5,6]. Sleep disordered breathing (SDB), defined as repetitive episodes of decrease or total cessation of respiratory airflow during sleep, which can be categorized into obstructive sleep apnea (OSA) or central sleep apnea (CSA), has been shown to closely interact with and be involved in the pathophysiology of heart disease [7,8,9,10,11]. Whilst OSA is known to correlate with cardiovascular morbidity and mortality [6]; CSA is commonly observed among patients with heart failure (HF) but its relationship to cardiovascular disease is not fully understood yet [6,9,12].

Interestingly, established treatment strategies of HF (e.g., cardiac resynchronization therapy (CRT) and valve repair) have been shown to improve SDB [6,13,14,15,16,17]. However, patients with mitral regurgitation who are at high risk for surgical intervention are candidates for less invasive percutaneous techniques such as the MitraClip system (Abbott Vascular, Abbott Park, IL, USA), which is currently the device with the widest clinical use [18,19,20]. Nevertheless, whilst some reports have shown that surgical repair of MR may result in a reduction in concomitant SDB [6,21,22], only a small case series suggested a similar benefit of MitraClip-placement [23]. Overall, data about the effects of MitraClip-placement on sleep quality and their clinical significance are very scarce.

Therefore, the aim of this study was to examine the prevalence of SDB among patients with MR undergoing MitraClip-procedure and to determine the impact of MitraClip-placement on SDB. We also aimed to analyze the patients´ QoL after MitraClip-placement in the light of changes in their sleep quality.

## 2. Materials and Methods

The protocol for this prospective study was approved by the local ethics committee, and the study was performed in accordance with the ethical standards laid down in the Declaration of Helsinki in its latest revision. Written informed consent was obtained from all patients prior to inclusion. The study was registered on www.clinicaltrials.gov, accesed on 1 June 2021 (NCT02615431).

**Figure 1:**
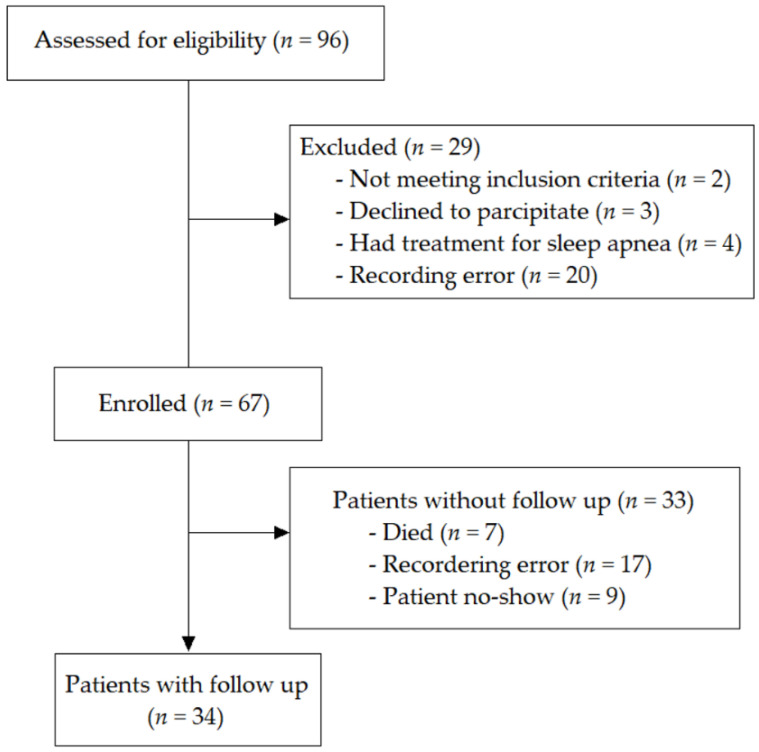
Consort flow chart showing how the analytical sample was derived from the participants who were assessed for eligibility.

Consecutive patients hospitalized at the University Hospital RWTH Aachen for MitraClip-procedure due to “moderate” (2+), through “moderate/severe” (3+), through “severe” (4+) mitral valve regurgitation were prospectively enrolled. MR severity was determined echocardiographically by primary investigators. All patients were already discussed and accepted by the heart team to undergo a percutaneous edge-to-edge mitral valve repair using the MitraClip system (Abbott Vascular, Abbott Park, IL, USA) [24]. Patients who were or had been on any treatment for sleep apnea were excluded (Figure 1).

Demographic data, disease history, coexisting medical conditions, smoking, and medication history were recorded for all patients. Furthermore, electrocardiography, transthoracic echocardiography, and blood sampling were performed. The validated European-System-for-Cardiac-Operative-Risk-Evaluation-SCORE II (EuroSCORE II), which estimates the risk of in-hospital death after cardiac surgery, was calculated for all patients [25,26]. Symptoms of daytime sleepiness were assessed using the Epworth Sleepiness Scale (ESS), which provides a simple standardized mean of quantifying daytime sleepiness, with scores above 10 consistent with the suspicion of SDB [27,28,29]. Furthermore, sleep quality was assessed using the Pittsburgh Sleep Quality Index (PSQI), which represents a subjective self-assessment of sleep quality. PSQI includes a total of 18 items assigned to seven components, each of which can assume a value range from 0 to 3, with the total score ranging from 0 to 21, and with lower scores denoting healthier sleep quality [30,31].

Before MitraClip-placement, all patients underwent SDB screening using five-channel respiratory polygraphy (ApneaLink™; ResMed, San Diego, CA, USA), which is an accurate and reliable device for the diagnosis of SDB [32,33]. The device records five signals: respiratory effort, pulse, oxygen saturation, nasal airflow, and snoring. The total number of apneas, hypopneas, and apnea-hypopnea-index (AHI) per hour recording time were documented. The determination of apneas/hypopneas and the calculation of AHI were performed according to the recommendations of the American Academy of Sleep Medicine (AASM) and to the Chicago consensus paper criteria [34,35,36]. We used the cutoff of AHI ≥ 15/h in the diagnosis of SDB, which is a highly sensitive, specific, and reliable criterion in the diagnosis of moderate to severe SDB according to the current guidelines [33,37,38]. Hypopneas were scored when all three of the following criteria were met [34]:

  - Airflow decreases at least 30 percent compared with the pre-event baseline,
  - The diminished airflow lasts at least 10 s,
  - The event is associated with a 3 percent oxygen desaturation from baseline.

Additionally, the ApneaLink software calculates risk indicator (RI), which is a combination of AHI plus inspiratory flow limitation events, and has similar diagnostic accuracy to AHI and is highly sensitive and specific to diagnose moderate to severe SDB (sensitivity 93.5% and specificity 91% by the cut-off point RI > 16) [33].

Furthermore, serum, plasma, and whole blood samples were obtained routinely at the time of admission. Complete blood count, circulating levels of C-reactive protein (CRP), N-terminal pro B-type natriuretic peptide (NT-proBNP), and creatinine levels in blood were measured.

All patients were invited for a follow-up examination. No SDB therapy was initiated. Patients had a reassessment similar to that at recruitment including: ESS, PSQI, electrocardiography, transthoracic echocardiography, blood sampling, and five-channel respiratory polygraphy (ApneaLink™Air).

The sample size was planned to detect a minimal difference of 10 points in AHI with an 80% probability using a type 1 error of 0.05, and a standard deviation of 20 points. The minimal number of participants was 34.

Assessed variables were described using frequencies and percentages for categorical variables and means, standard deviations, quartiles, and minimum, maximum variables depending on the distributions for continuous variables. Variables were described by AHI categories (<15/h, ≥15/h), and differences from baseline for follow-up assessments were calculated and described. Explorative comparisons between AHI groups were performed using Kruskal–Wallis test and Pearson Chi-squared test. Baseline and follow-up measurements were compared using Wilcoxon signed rank test.

## 3. Results

There were 67 patients (age 77 ± 8 years, 58% male) included. Baseline data and comorbidities are described in Table 1.

Pre-procedural scores and measurements and characteristics of mitral valve regurgitation are described in Table 2. The ESS values were mostly normal (median 5 (interquartile range IQR: 2–7)), and PSQI values were also within normal range (8 ± 4.1) (Table 2).

However, 41 patients (61%) had an AHI ≥ 15/h (Table 3). Only 3 of these 41 patients had been previously diagnosed with SDB (not treated). The vast majority of patients suffered predominantly from OSA (37 patients), and only 4 patients suffered from CSA (two of them had predominant Cheyne–Stokes respiration (CSR) pattern) (Table 3).

Comparing patients with and without SDB at baseline, there was no substantial difference in left ventricular ejection fraction (LVEF), MR-grade, or other characteristics of MR and echocardiographic variables (Table 4). EuroSCORE and sleepiness scores were also not significantly different at baseline between both groups (Table 4). However, patients with AHI ≥ 15/h had higher NT-proBNP values compared to patients with an AHI < 15/h (median = 4325 pg/mL (IQR: 2823–7368) vs. 1520 (437–2955) respectively, *p* < 0.001) (Table 4).

**Figure 2:**
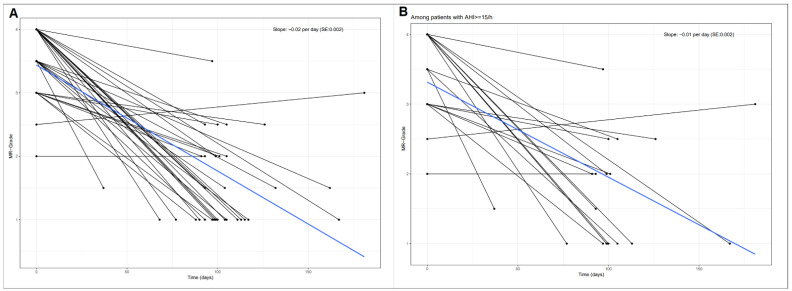
Progression of mitral valve regurgitation grade during follow-up in 34 patients. (**A**) In all patients (*p* < 0.001). (**B**) In patients with baseline-AHI ≥ 15 (*p* < 0.001).

Of the 67 patients, 34 patients were admitted for a follow-up examination after MitraClip-placement (mean follow-up time: 105 ± 27 days, median: 100 days after MitraClip-placement). There was a clear improvement of MR-grade in most patients (Figure 2).

**Figure 3:**
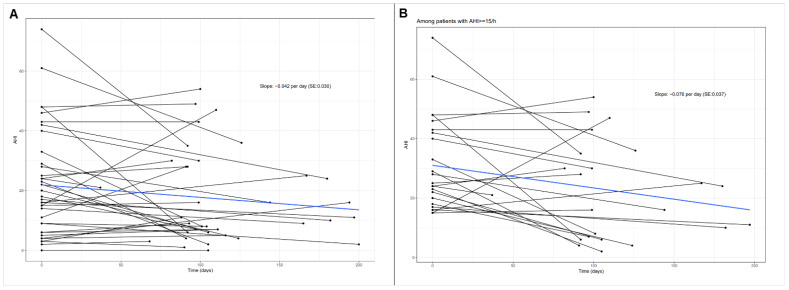
Progression of apnea-hypopnea-index (AHI) during follow-up in 34 patients. (**A**) In all patients (*p* = 0.077). (**B**) In patients with baseline-AHI ≥ 15 (*p* = 0.013).

The median difference in MR-grade from baseline was −2.5 (IQR: −3.0–−1.5; *p* < 0.001) on a severity scale of +1 to +4. LVEF and other echocardiographic variables did not change over time. Importantly, there were improvements in AHI (*p* = 0.077), which reached statistical significance in patients with pre-interventional AHI ≥ 15/h (*p* = 0.013) (Figure 3).

**Figure 4:**
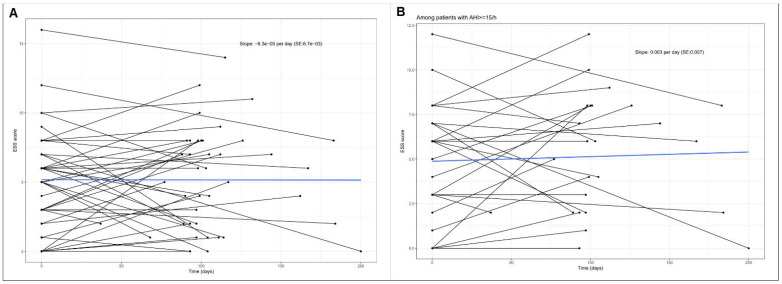
Progression of Epworth Sleepiness Scale (ESS) during follow-up in 34 patients. (**A**) In all patients (*p* = 0.999). (**B**) In patients with baseline-AHI ≥ 15 (*p* = 0.636).

Changes in AHI did not correlate with changes in LVEF (Pearson´s r = −0.13, *p* = 0.516) even in patients with pre-interventional AHI ≥ 15/h (Pearson´s r = −0.10, *p* = 0.739). There were no significant changes in the ESS score over time (Figure 4).

The major outcomes before and after the procedure are presented in Table 5.

## 4. Discussion

The present study demonstrates that the prevalence of SDB in patients undergoing MitraClip-placement is very high even in non-sleepy patients and is therefore underestimated if the diagnosis of SDB is based on sleepiness scores only. Secondly, patients with SDB had significantly higher levels of NT-proBNP at baseline, which may reflect a worse prognosis in these patients. Thirdly, there was an obvious reduction in AHI at follow-up after MitraClip-placement among those with SDB, which could be due to many cooperating confounders, but also might still be an additional benefit of the MitraClip procedure.

According to epidemiological data, 2–7% of adults suffer from OSA, and the prevalence in patients with cardiovascular diseases is 2–3 times higher compared to the general population [39,40]. Furthermore, in patients with HF and preserved left ventricular function (HFpEF), CSA is found in approximately 18 to 30% [41,42,43], and although the relationship between CSA and cardiovascular disease is not fully understood yet, CSA may hypothetically expose the already failing heart to hypoxia, thus contributing to worsening heart function by sympathetic nervous system activation and ventricular arrhythmias [6,9]. Nevertheless, there is not enough data about the prevalence of SDB in patients with valvular heart disease. In our cohort, 62% of all patients with moderate to severe MR had SDB which is considerably higher compared to the last-mentioned patient groups with other cardiovascular comorbidities. However, candidates for MitraClip-placement represent a distinct subgroup of patients with MR, who do not only suffer from systolic HF but are also advanced in age, have reduced functional status, have more cardiovascular and other co-morbidities reflected by the high EuroSCORE-II in our cohort. The latter constellation may represent a logical explanation for the high prevalence of SDB in our patient population since the prevalence of SDB is known to increase with age [39,40].

Importantly, the ESS score was not increased in patients with SDB. In fact, there is strong evidence that SDB frequently occurs in non-sleepy HF patients associated with increased morbidity and mortality [41,44,45]. Making the diagnosis of SDB in the pre-procedural setting is of high importance, as SDB is a frequently missed diagnosis associated with an increased risk of various peri-interventional complications when sedation or general anesthesia is used [46,47,48,49,50,51]. Pre-interventional diagnosis and treatment of SDB might at least partially reduce the risk for complications [52,53]. Hence, integrating systematic screening for SDB in the pre-interventional assessment of patients undergoing MitraClip-placement, even in those without daytime sleepiness, could be an interesting and beneficial approach, as patients with SDB require more peri-procedural attention and a specific anesthetic approach and might even profit from the initiation of continuous positive airway pressure therapy prior to the procedure [38]. However, while sleepiness scores may underestimate SDB in non-sleepy populations, it should be noted that non-sleepy elderly participants may not benefit from treatment of OSA, and/or CSA, other than with the usual management of heart failure.

Patients with SDB in our cohort had higher values of NT-proBNP before MitraClip-placement than those without SDB, although echocardiographic and renal parameters were comparable between both groups. Since elevated plasma NT-proBNP levels, as well as the presence of SDB, are associated with an unfavorable prognosis in patients suffering from different heart diseases [54,55], the latter finding could indicate a worse prognosis in this group of patients. On the other hand, this finding suggests that NT-proBNP could serve as a pre-interventional biomarker for the detection of SDB in MR patients. Detecting and management of SDB in this group of patients may be of clinical importance, since SDB management is known to reduce cardiac risk [56], along with improvements in LVEF [57], QoL, morbidity, and mortality rates [58]. However, this needs further investigation.

On the other hand, it has been shown that guideline-directed treatment of HF can improve or even eliminate CSA [14,16,59,60]. Among patients with SDB in our cohort, a significant reduction in AHI was observed at the follow-up visits 3–6 months after MitraClip-placement. This improvement may facilitate positive effects on QoL and prognosis in patients with MR undergoing MitraClip-placement. Improvements in nocturnal or supine nasopharyngeal congestion, which might be caused by mitral regurgitation, and could be reduced through MitraClip-placement; seems a very logical explanation of these improvements after MitraClip-placement, as the fluid shifts to the upper airway in the recumbent position, may cause the pharynx to become narrowed and more susceptible to collapse [61,62]. The fact that many patients in our cohort had OSA would support this speculation, since OSA may well be improved by decreasing nasopharyngeal congestion through MitraClip. However, this needs further investigation in future studies.

Our study has some limitations which need to be addressed. Firstly, we used five channel respiratory polygraphy in evaluating SDB instead of polysomnography. This approach does not allow the documentation of arousal events. Furthermore, we used an automated scoring alone without review by an accredited sleep scientist; and oxygen related parameters (e.g., the proportion of the sleep time with an SpO2 < 90% (T90%) and oxygen desaturation index (ODI)) were not registered. Besides that, the diagnostic performance of the ESS (>10) for predicting SDB has suboptimal sensitivity and specificity, and several screening questionnaires have been developed for preoperative screening that incorporates risk factors, clinical symptoms, and physical examination parameters (e.g., Berlin and STOP-BANG questionnaires), which were not used in our study. Nevertheless, the used respiratory polygraphy device has an adequate selection of bio-signals and allows—in the presence of good signal acquisition and processing—an accurate diagnosis of sleep disorders [39,63]. Secondly, due to the observational non-interventional design of our study, there was no control group of patients with medical therapy only. Therefore, future studies should focus on the bidirectional effects of sleep disorders and valvular heart disease and their respective treatments. Thirdly, a significant number of patients were lost for follow-up. Therefore, it cannot be excluded that this might have influenced the outcome measures. Lastly, other confounders may have led to changes in SDB at follow-up (e.g., volume status, weight changes, and/or optimization of other heart disease treatments). However, it is unlikely that these factors strongly influenced the results, because all medical therapy options and optimization of risk factors were already tapped before MitraClip-placement.

## 5. Conclusions

In conclusion, the prevalence of sleep disordered breathing among patients with MR undergoing MitraClip-placement is very high even in non-sleepy patients. MR Patients with SDB have higher NT-proBNP levels, which may reflect a worse prognosis in these patients. Therefore, screening patients undergoing MitraClip-placement for SDB might be of clinical importance, especially those with high NT-proBNP levels. Finally, MitraClip-placement seems to improve the underlying sleep disorder in patients with moderate to severe SDB, which may be an additional benefit from MitraClip-procedure.

## Figures and Tables

**Table 1:** Patient clinical and demographic data at baseline.

	Reference Values	Patients (*n* = 67)
Age, years		77 ± 8
Male		39 (58%)
BMI (kg/m^2^)		25.6 ± 4.7
Smoking history		
- Current smoker		5 (7%)
- Ex-smoker		21 (31%)
- Never smoked		40 (60%)
Comorbidities at admission		
- Coronary heart disease		44 (66%)
- Previous myocardial infarction		16 (24%)
- Coronary artery bypass surgery		12 (18%)
- Peripheral artery disease		5 (7%)
- Chronic heart failure		37 (55%)
- NYHA II		7 (10%)
- NYHA III		42 (63%)
- NYHA IV		18 (27%)
- Recent decompensated heart failure (last 3 Months)		27 (40%)
- Dilated cardiomyopathy		24 (36%)
- Hypertension		48 (72%)
- Diabetes mellitus		21 (31%)
- Chronic kidney disease		34 (51%)
- Cerebrovascular disease		9 (13%)
- Atrial fibrillation		39 (58%)
- COPD		15 (22%)
- Cardiac pacemaker/ICD/CRT		22 (33%)
- Known sleep apnea		4 (6%)
Medications		
- ACE-I		36 (54%)
- ß-Blocker		42 (63%)
- Aldosterone antagonist		20 (30%)
- Loop diuretics		49 (73%)
Laboratory tests		
- NT-proBNP, pg/mL	<220	3056 [1571;5395]
- CRP, mg/dL	<5	6 [2;12]
- Creatinine, mg/dL	0.5–1.2	1.5 ± 0.63
- GFR, mL/min/1.73 m^2^	≥60	48 ± 20.2

Values are presented as mean ± standard deviation, number of patients (percentage) or median [interquartile range]. ACE-I, Angiotensin converting enzyme inhibitor; BMI, body mass index; COPD, chronic obstructive pulmonary disease; CRP, C-reactive protein; CRT, cardiac resynchronization therapy; GFR, glomerular filtration rate; ICD, implantable cardioverter defibrillator; NT-proBNP, N-terminal pro B-type natriuretic peptide; NYHA, New York Heart Association.

**Table 2:** Pre-procedural scores and measurements, characteristics of mitral valve regurgitation, and peri-procedural data of MitraClip intervention.

	Patients (*n* = 67)
Preprocedural scores and measurements	
- EuroSCORE II	7 ± 4.1
- Epworth Sleepiness Scale (ESS)	5 [2;7]
- Total Pittsburgh Sleep Quality Index (PSQI)	8 ± 4.1
Characteristics of mitral valve regurgitation	
Etiology of the MR	
- degenerative, *n* (%)	18 (27%)
- functional, *n* (%)	44 (66%)
- mixed, *n* (%)	5 (7%)
MR grade	4+ [3+;4+]
Echocardiography	
- LVEF, % (normal: 55–70%)	39 ± 14
- EROA, mm^2^	33 ± 13
- Regurgitation volume, mL	53 ± 18
- PISA, mm	8.8 ± 1.98
- Pap_sys_, mmHg (normal: ≤35)	47 ± 14
Procedural and postprocedural data of MitraClip	
- Number of clips, *n*	2 [1;2]
- Difference in MR grade directly after procedure	−2.5 [−3.0;−1.5]
- Total days in hospital, *n*	13 [7;21]
- Pap_mean_ after MitraClip, mmHg	3.0 [3;5]
- In-Hospital MACCE, *n* (%)	0 (0%)

Values are presented as mean ± standard deviation, number of patients (percentage), or median [interquartile range]. EROA, effective regurgitant orifice area; EuroSCORE, European-System-for-Cardiac-Operative-Risk-Evaluation-SCORE; LVEF, left ventricular ejection fraction; MACCE, major adverse cardiac and cerebrovascular events; MR, mitral regurgitation; Papmean, mean pulmonary artery pressure; Papsys, systolic pulmonary artery pressure; PISA, proximal isovelocity surface area.

**Table 3:** Respiratory polygraphy in 67 patients.

	Patients (*n* = 67)
AHI, /h	18 [6;37]
Risk indicator (RI)	21 [8;38]
Patients with SDB (AHI ≥ 15)	41 (62%)
- Patients with CSA, *n* (%)	4 (6%)
- Patients with OSA, *n* (%)	37 (55%)
Proportion Cheyne–Stokes epochs, % of the whole CSA epochs	2 [0;14]

Values are presented as mean ± standard deviation, number of patients (percentage), or median [interquartile range]. AHI, apnea-hypopnea-index; CSA, central sleep apnea; OSA, obstructive sleep apnea; SDB, sleep disordered breathing.

**Table 4:** Comparison of patients with versus without sleep disordered breathing (SDB).

	Reference Values	AHI < 15/h (*n* = 26)	AHI ≥ 15/h (*n* = 41)	*p*-Value
Age, years		74 ± 8	79 ± 7	0.005
Male		12 (46%)	27 (66%)	0.181
BMI, kg/m^2^		25 ± 5	26 ± 5	0.126
Laboratory tests				
- NT-proBNP, pg/mL	<220	1520 [437;2955]	4325 [2823;7368]	<0.001
- CRP, mg/dL	<5	6 [3;7]	6 [2;15]	0.661
- Creatinine, mg/dL	0.5–1.2	1.4 ± 0.55	1.5 ± 0.68	0.639
- GFR, mL/min/1.73 m^2^	≥60	50 ± 21	47 ± 20	0.629
Scores and measurements				
- EuroSCORE I		20 ± 11	23 ± 11	0.338
- EuroSCORE II		6 ± 3.8	8 ± 4.3	0.254
- ESS		5 [2;8]	5 [2;7]	0.856
- PSQI		8 ± 4.5	9 ± 4.0	0.745
Characteristics of MR				
Etiology of the MR				
- degenerative, *n* (%)		8 (31%)	10 (24%)	0.760
- functional, *n* (%)		17 (65%)	27 (68%)	0.760
- mixed, *n* (%)		1 (4%)	3 (8%)	0.760
MR grade		4+ [3+;4+]	4+ [3+;4+]	0.612
Mitraclip^®^ procedure				
- Number of clips, *n*		1 (1,2)	2 (1,2)	0.061
- Difference in MR-grade directly after MitraClip		−2.5 [−3.0;−1.5]	−2.5 [−2.6;−1.5]	0.888
- Total days in hospital, *n*		13 [7;20]	14 [9;21]	0.437
- Pap_mean_ after MitraClip, mmHg		3 [3;5]	3 [2;5]	0.305
- In-Hospital MACCE, *n* (%)		0 (0%)	0 (0%)	-
Respiratory polygraphy (Apnea Link)				
- AHI, /h		5 [3;8]	22 [33;46]	<0.001
- Risk indicator (RI)		8 [6;10]	33 [23;47]	<0.001

Values are presented as mean ± standard deviation, number of patients (percentage), or median [interquartile range]. AHI, apnea-hypopnea-index; BMI, body mass index; CRP, C-reactive protein; CSA, central sleep apnea; ESS, Epworth Sleepiness Scale; EuroSCORE, European-System-for-Cardiac-Operative-Risk-Evaluation-SCORE; GFR, glomerular filtration rate; MACCE, major adverse cardiac and cerebrovascular events; MR, mitral regurgitation; NT-proBNP, N-terminal pro B-type natriuretic peptide; OSA, obstructive sleep apnea; Papmean, mean pulmonary artery pressure; PSQI, Pittsburgh Sleep Quality Index; SDB, sleep disordered breathing.

**Table 5:** Comparison of outcomes before and after MitraClip in 34 patients with follow-up data.

	Before MitraClip	After MitraClip
AHI, /h	18 [7;33]	11 [6;29]
ESS	5 [2;8]	5 [2;8]
PSQI	7 [5;11]	7 [4;11]
NT-proBNP, pg/mL	2990 [1503;7236]	3348 [1723;5099]

Values are presented as median [interquartile range]. AHI, apnea-hypopnea-index; ESS, Epworth Sleepiness Scale; NT-proBNP, N-terminal pro B-type natriuretic peptide; PSQI, Pittsburgh Sleep-Quality Index.

## Data Availability

Data are available upon reasonable request to the corresponding author.

## References

[1] Gaasch W.H., Otto C.M., Post T.W. (2021). Pathophysiology and Natural History of Chronic Mitral Regurgitation.

[2] Nishimura R.A., Otto C.M., Bonow R.O., Carabello B.A., Erwin J.P., Guyton R.A., O’Gara P.T., Ruiz C.E., Skubas N.J., Sorajja P. (2014). 2014 AHA/ACC guideline for the management of patients with valvular heart disease: A report of the American College of Cardiology/American Heart Association Task Force on Practice Guidelines. J. Am. Coll. Cardiol..

[3] Stone G.W., Lindenfeld J., Abraham W.T., Kar S., Lim D.S., Mishell J.M., Whisenant B., Grayburn P.A., Rinaldi M., Kapadia S.R. (2018). Transcatheter Mitral-Valve Repair in Patients with Heart Failure. N. Engl. J. Med..

[4] Parati G., Lombardi C., Castagna F., Mattaliano P., Filardi P.P., Agostoni P. (2016). Heart failure and sleep disorders. Nat. Rev. Cardiol..

[5] Jean-Louis G., Zizi F., Clark L.T., Brown C.D., McFarlane S.I. (2008). Obstructive sleep apnea and cardiovascular disease: Role of the metabolic syndrome and its components. J. Clin. Sleep Med..

[6] Abe H., Takahashi M., Yaegashi H., Eda S., Kitahara H., Tsunemoto H., Kamikozawa M., Koyama J., Yamazaki K., Ikeda U. (2009). Valve repair improves central sleep apnea in heart failure patients with valvular heart diseases. Circ. J. Off. J. Jpn. Circ. Soc..

[7] Young T., Palta M., Dempsey J., Skatrud J., Weber S., Badr S. (1993). The occurrence of sleep-disordered breathing among middle-aged adults. N. Engl. J. Med..

[8] Parish J.M., Somers V.K. (2004). Obstructive sleep apnea and cardiovascular disease. Mayo Clin. Proc..

[9] Bradley T.D., Floras J.S. (2003). Sleep Apnea and Heart Failure. Circulation.

[10] Ferrier K., Campbell A., Yee B., Richards M., O’Meeghan T., Weatherall M., Neill A. (2005). Sleep-disordered breathing occurs frequently in stable outpatients with congestive heart failure. Chest.

[11] White D.P. (2005). Pathogenesis of obstructive and central sleep apnea. Am. J. Respir. Crit. Care Med..

[12] Vazir A., Hastings P.C., Dayer M., McIntyre H.F., Henein M.Y., Poole-Wilson P.A., Cowie M.R., Morrell M.J., Simonds A.K. (2007). A high prevalence of sleep disordered breathing in men with mild symptomatic chronic heart failure due to left ventricular systolic dysfunction. Eur. J. Heart Fail..

[13] Gabor J.Y., Newman D.A., Barnard-Roberts V., Korley V., Mangat I., Dorian P., Hanly P.J. (2005). Improvement in Cheyne-Stokes respiration following cardiac resynchronisation therapy. Eur. Respir. J..

[14] Sinha A.M., Skobel E.C., Breithardt O.A., Norra C., Markus K.U., Breuer C., Hanrath P., Stellbrink C. (2004). Cardiac resynchronization therapy improves central sleep apnea and Cheyne-Stokes respiration in patients with chronic heart failure. J. Am. Coll. Cardiol..

[15] Skobel E.C., Sinha A.M., Norra C., Randerath W., Breithardt O.A., Breuer C., Hanrath P., Stellbrink C. (2005). Effect of cardiac resynchronization therapy on sleep quality, quality of life, and symptomatic depression in patients with chronic heart failure and Cheyne-Stokes respiration. Sleep Breath. Schlaf Atm..

[16] Oldenburg O., Faber L., Vogt J., Dorszewski A., Szabados F., Horstkotte D., Lamp B. (2007). Influence of cardiac resynchronisation therapy on different types of sleep disordered breathing. Eur. J. Heart Fail..

[17] Kara T., Novak M., Nykodym J., Bybee K.A., Meluzin J., Orban M., Novakova Z., Lipoldova J., Hayes D.L., Soucek M. (2008). Short-term effects of cardiac resynchronization therapy on sleep-disordered breathing in patients with systolic heart failure. Chest.

[18] Wan B., Rahnavardi M., Tian D.H., Phan K., Munkholm-Larsen S., Bannon P.G., Yan T.D. (2013). A meta-analysis of MitraClip system versus surgery for treatment of severe mitral regurgitation. Ann. Cardiothorac. Surg..

[19] Ray R., Chambers J. (2014). Mitral valve disease. Int. J. Clin. Pract..

[20] Condado J.A., Vélez-Gimón M. (2003). Catheter-based approach to mitral regurgitation. J. Interv. Cardiol..

[21] Takahashi M., Kasai T., Dohi T., Maeno K.-i., Kasagi S., Kawana F., Ishiwata S., Narui K. (2011). Conversion from predominant central sleep apnea to obstructive sleep apnea following valvuloplasty in a patient with mitral regurgitation. J. Clin. Sleep Med..

[22] Rubin A.E., Gottlieb S.H., Gold A.R., Schwartz A.R., Smith P.L. (2004). Elimination of central sleep apnoea by mitral valvuloplasty: The role of feedback delay in periodic breathing. Thorax.

[23] Spiesshoefer J., Spieker M., Klose S., Keymel S., Boentert M., Krüger S., Horn P., Kelm M., Westenfeld R. (2019). Reduction of sleep-disordered breathing following effective percutaneous mitral valve repair with the MitraClip system. Sleep Breath. Schlaf Atm..

[24] Kelley C., Lazkani M., Farah J., Pershad A. (2016). Percutaneous mitral valve repair: A new treatment for mitral regurgitation. Indian Heart J..

[25] Nashef S.A., Roques F., Michel P., Gauducheau E., Lemeshow S., Salamon R. (1999). European system for cardiac operative risk evaluation (EuroSCORE). Eur. J. Cardio-Thorac. Surg..

[26] Nashef S.A., Roques F., Sharples L.D., Nilsson J., Smith C., Goldstone A.R., Lockowandt U. (2012). EuroSCORE II. Eur. J. Cardio-Thorac. Surg..

[27] Johns M.W. (1991). A new method for measuring daytime sleepiness: The Epworth sleepiness scale. Sleep.

[28] Johns M.W. (2000). Sensitivity and specificity of the multiple sleep latency test (MSLT), the maintenance of wakefulness test and the epworth sleepiness scale: Failure of the MSLT as a gold standard. J. Sleep Res..

[29] Doneh B. (2015). Epworth Sleepiness Scale. Occup. Med..

[30] Buysse D.J., Reynolds C.F., Monk T.H., Berman S.R., Kupfer D.J. (1989). The Pittsburgh Sleep Quality Index: A new instrument for psychiatric practice and research. Psychiatry Res..

[31] Grandner M.A., Kripke D.F., Yoon I.-Y., Youngstedt S.D. (2006). Criterion validity of the Pittsburgh Sleep Quality Index: Investigation in a non-clinical sample. Sleep Biol. Rhythm..

[32] Cho J.H., Kim H.J. (2017). Validation of ApneaLink™ Plus for the diagnosis of sleep apnea. Sleep Breath. Schlaf Atm..

[33] Nigro C.A., Serrano F., Aimaretti S., González S., Codinardo C., Rhodius E. (2010). Utility of ApneaLink for the diagnosis of sleep apnea-hypopnea syndrome. Medicina.

[34] Iber C., Ancoli-Israel S., Chesson A.L., Quan S.F. (2007). The AASM Manual for the Scoring of Sleep and Associated Events: Rules, Terminology and Technical Specifications.

[35] Berry R.B., Budhiraja R., Gottlieb D.J., Gozal D., Iber C., Kapur V.K., Marcus C.L., Mehra R., Parthasarathy S., Quan S.F. (2012). Rules for scoring respiratory events in sleep: Update of the 2007 AASM Manual for the Scoring of Sleep and Associated Events. Deliberations of the Sleep Apnea Definitions Task Force of the American Academy of Sleep Medicine. J. Clin. Sleep Med..

[36] Quan S.F., Gillin J.C., Littner M.R., Shepard J.W. (1999). Sleep-related breathing disorders in adults: Recommendations for syndrome definition and measurement techniques in clinical research. The Report of an American Academy of Sleep Medicine Task Force. Sleep.

[37] Gerlach M., Sanner B. (2017). Guidelines in Practice: The New S3 Guideline “Sleeping Disorders—Sleep-Related Abnormal Breathing”. Pneumologie.

[38] Riemann D., Baum E., Cohrs S., Crönlein T., Hajak G., Hertenstein E., Klose P., Langhorst J., Mayer G., Nissen C. (2017). S3-Leitlinie Nicht erholsamer Schlaf/Schlafstörungen—Kapitel Schlafbezogene Atmungsstörungen. Somnologie.

[39] Collop N.A., Tracy S.L., Kapur V., Mehra R., Kuhlmann D., Fleishman S.A., Ojile J.M. (2011). Obstructive sleep apnea devices for out-of-center (OOC) testing: Technology evaluation. J. Clin. Sleep Med..

[40] Young T., Peppard P.E., Gottlieb D.J. (2002). Epidemiology of obstructive sleep apnea: A population health perspective. Am. Respir. Crit. Care Med..

[41] Chan J., Sanderson J., Chan W., Lai C., Choy D., Ho A., Leung R. (1997). Prevalence of sleep-disordered breathing in diastolic heart failure. Chest.

[42] Herrscher T.E., Akre H., Øverland B., Sandvik L., Westheim A.S. (2011). High prevalence of sleep apnea in heart failure outpatients: Even in patients with preserved systolic function. J. Card. Fail..

[43] Sekizuka H., Osada N., Miyake F. (2013). Sleep disordered breathing in heart failure patients with reduced versus preserved ejection fraction. Heart Lung Circ..

[44] Javaheri S., Shukla R., Zeigler H., Wexler L. (2007). Central sleep apnea, right ventricular dysfunction, and low diastolic blood pressure are predictors of mortality in systolic heart failure. J. Am. Coll. Cardiol..

[45] Lanfranchi P.A., Braghiroli A., Bosimini E., Mazzuero G., Colombo R., Donner C.F., Giannuzzi P. (1999). Prognostic value of nocturnal Cheyne-Stokes respiration in chronic heart failure. Circulation.

[46] Vasu T.S., Grewal R., Doghramji K. (2012). Obstructive sleep apnea syndrome and perioperative complications: A systematic review of the literature. J. Clin. Sleep Med..

[47] Kim J.A., Lee J.J. (2006). Preoperative predictors of difficult intubation in patients with obstructive sleep apnea syndrome. Can. J. Anesth..

[48] Siyam M.A., Benhamou D. (2002). Difficult endotracheal intubation in patients with sleep apnea syndrome. Anesth. Analg..

[49] Stierer T.L., Wright C., George A., Thompson R.E., Wu C.L., Collop N. (2010). Risk assessment of obstructive sleep apnea in a population of patients undergoing ambulatory surgery. J. Clin. Sleep Med..

[50] Kaw R., Pasupuleti V., Walker E., Ramaswamy A., Foldvary-Schafer N. (2012). Postoperative complications in patients with obstructive sleep apnea. Chest.

[51] Finkel K.J., Searleman A.C., Tymkew H., Tanaka C.Y., Saager L., Safer-Zadeh E., Bottros M., Selvidge J.A., Jacobsohn E., Pulley D. (2009). Prevalence of undiagnosed obstructive sleep apnea among adult surgical patients in an academic medical center. Sleep Med..

[52] Gupta R.M., Parvizi J., Hanssen A.D., Gay P.C. (2001). Postoperative complications in patients with obstructive sleep apnea syndrome undergoing hip or knee replacement: A case-control study. Mayo Clin. Proc..

[53] Mutter T.C., Chateau D., Moffatt M., Ramsey C., Roos L.L., Kryger M. (2014). A matched cohort study of postoperative outcomes in obstructive sleep apnea: Could preoperative diagnosis and treatment prevent complications?. Anesthesiology.

[54] Chen A.A., Wood M.J., Krauser D.G., Baggish A.L., Tung R., Anwaruddin S., Picard M.H., Januzzi J.L. (2006). NT-proBNP levels, echocardiographic findings, and outcomes in breathless patients: Results from the ProBNP Investigation of Dyspnoea in the Emergency Department (PRIDE) echocardiographic substudy. Eur. Heart J..

[55] Drager L.F., McEvoy R.D., Barbe F., Lorenzi-Filho G., Redline S. (2017). Sleep Apnea and Cardiovascular Disease: Lessons From Recent Trials and Need for Team Science. Circulation.

[56] Lévy P., Kohler M., McNicholas W.T., Barbé F., McEvoy R.D., Somers V.K., Lavie L., Pépin J.L. (2015). Obstructive sleep apnoea syndrome. Nature reviews. Dis. Primers.

[57] Sun H., Shi J., Li M., Chen X. (2013). Impact of continuous positive airway pressure treatment on left ventricular ejection fraction in patients with obstructive sleep apnea: A meta-analysis of randomized controlled trials. PLoS ONE.

[58] Ayas N.T., FitzGerald J.M., Fleetham J.A., White D.P., Schulzer M., Ryan C.F., Ghaeli R., Mercer G.W., Cooper P., Tan M.C.Y. (2006). Cost-effectiveness of Continuous Positive Airway Pressure Therapy for Moderate to Severe Obstructive Sleep Apnea/Hypopnea. Arch. Intern. Med..

[59] Mansfield D.R., Solin P., Roebuck T., Bergin P., Kaye D.M., Naughton M.T. (2003). The effect of successful heart transplant treatment of heart failure on central sleep apnea. Chest.

[60] Solin P., Bergin P., Richardson M., Kaye D.M., Walters E.H., Naughton M.T. (1999). Influence of pulmonary capillary wedge pressure on central apnea in heart failure. Circulation.

[61] Shepard J.W., Pevernagie D.A., Stanson A.W., Daniels B.K., Sheedy P.F. (1996). Effects of changes in central venous pressure on upper airway size in patients with obstructive sleep apnea. Am. J. Respir. Crit. Care Med..

[62] Bradley T.D., Floras J.S. (2003). Sleep apnea and heart failure: Part I: Obstructive sleep apnea. Circulation.

[63] Collop N.A., Anderson W.M., Boehlecke B., Claman D., Goldberg R., Gottlieb D.J., Hudgel D., Sateia M., Schwab R. (2007). Clinical guidelines for the use of unattended portable monitors in the diagnosis of obstructive sleep apnea in adult patients. Portable Monitoring Task Force of the American Academy of Sleep Medicine. J. Clin. Sleep Med..

